# Functional Connectivity fMRI of the Rodent Brain: Comparison of Functional Connectivity Networks in Rat and Mouse

**DOI:** 10.1371/journal.pone.0018876

**Published:** 2011-04-18

**Authors:** Elisabeth Jonckers, Johan Van Audekerke, Geofrey De Visscher, Annemie Van der Linden, Marleen Verhoye

**Affiliations:** Bio-Imaging Lab, University of Antwerp, Antwerp, Belgium; Tokyo Medical and Dental University, Japan

## Abstract

At present, resting state functional MRI (rsfMRI) is increasingly used in human neuropathological research. The present study aims at implementing rsfMRI in mice, a species that holds the widest variety of neurological disease models. Moreover, by acquiring rsfMRI data with a comparable protocol for anesthesia, scanning and analysis, in both rats and mice we were able to compare findings obtained in both species. The outcome of rsfMRI is different for rats and mice and depends strongly on the applied number of components in the Independent Component Analysis (ICA). The most important difference was the appearance of unilateral cortical components for the mouse resting state data compared to bilateral rat cortical networks. Furthermore, a higher number of components was needed for the ICA analysis to separate different cortical regions in mice as compared to rats.

## Introduction

The interest in resting state functional Magnetic Resonance Imaging (rsfMRI), a method commonly used to study functional connectivity in the brain has recently shown a marked increase and opened an interesting and growing avenue of investigations. In contrast to regular fMRI this technique does not require the subject to be stimulated or to perform a task while in the scanner. RsfMRI, measured during rest instead, aims at detecting low frequency fluctuations (LFFs) of less than 0.1 Hz in the Blood Oxygen level Dependent (BOLD) signal. Functional connectivity is defined here as temporal correlation of these fluctuations between different brain regions [1]. Functional communication between brain regions plays a key role in complex cognitive processes. Consequently, the examination of functional connectivity in the human brain is of high importance because it could provide new and important insights into the organization of the human brain [2] and reorganization during disease, learning and aging [3].

The concept of measuring the brain's resting state became popular in human research and different resting state networks have been defined since. The observed networks could be reproducibly distinguished both intra- and inter-individually [4]. These observations motivated a lot of interesting studies, assessing possible functional disconnectivity effects in both neurologic and psychiatric brain disorders [2], depression [5], dementia [6] and schizophrenia [7]. Consequently, rsfMRI became a very attractive candidate for defining (early) disease biomarkers as it is non-invasive, undemanding for the patient and limited in scanning time.

Notwithstanding several interesting clinical findings, a lot still remains to be discovered about the underlying processes responsible for the LFFs. The true neuronal basis of these low frequency rsfMRI oscillations is not yet fully understood. In the past years there has been an ongoing debate on the influence of physiological processes, like respiratory and cardiac oscillations [8] on the signal measured during rest originating from co-activation in the underlying spontaneous neuronal activation patterns of brain regions, measured through a hemodynamic response function [9].

Although rsfMRI experiments on animals are still scarce, limited only to rats and monkeys [10]–[24], they clearly have the potential to give more insight and understanding of the technique. Animal models offer the possibility to experimentally modify the functional connectivity with drugs and/or through disease modelling. Additionally, functional connectivity measurements could contribute in treatment efficacy studies. In other words, application of the technique in animal models clearly creates multiple opportunities either in using animal models and pharmaceutical compounds to investigate the technique, or either in using the technique to investigate pathologies and potential treatment regimes.

It should be mentioned that a lot of human resting state fMRI research is concentrated on the default mode network, however also other networks were included in these studies and changes in their functional connectivity are reported in several pathologies. For example in amyotrophic lateral sclerosis, a changed functional connectivity of the sensori-motor network is found [25] and in schizophrenia, the functional connections of the hippocampus are decreased [26]. Moreover, from research established in rats it was already known that the functional connectivity in cortical networks also could be modulated. It was shown that the interhemispheric functional connectivity for both motor cortex as somatosensory cortex was changed following limb deafferentation [24] and after stroke a changed interhemispheric functional connectivity for the somatosensory cortex was shown [13].

One rsfMRI processing technique used to estimate functional connectivity in human studies is a data-driven method called Independent Component Analysis (ICA) [27]. ICA divides the BOLD signal into different independent sources, or components. The fluctuations of the BOLD signal of all voxels of one component are temporally correlated. In other words, voxels of one component represent regions that are considered functionally connected. ICA allows data analysis without prior knowledge and gives the opportunity to investigate functional connectivity of the entire brain, making it more appropriate to investigate pathological influences on brain connectivity. The application of this technique on rat rsfMRI data has recently been reported [14].

The aim of our study was to implement rsfMRI ICA in mice and to compare these ICA derived functional connectivity maps between rats and mice. Although rats and mice are similar commonly used lab animals, their difference in size and physiology, i.e. breathing and heart rate, requires an adaptation of both anaesthesia as well as the scanning protocol. Moreover, only few studies exist reporting task based fMRI in mice [28]–[30], compared to a much higher amount of rat fMRI studies [31], showing that regular stimulation based fMRI is difficult to perform in mice. Proving that there are much less problems to obtain promising data by using resting state fMRI creates opportunities for a lot of interesting studies. Implementation of the rsfMRI technique with ICA in mice, which to our knowledge has not yet been reported, would clearly create the opportunity to study in a translational manner a plethora of mice models for different neuropathologies including the vast amount of transgenic mice currently available.

## Results

### Rat rsfMRI data

The rat data served as starting point for each comparison. The number of components used for this analysis was based on visual correlation of the different components to anatomically meaningful networks, without splitting up converging regions over different components or compiling non-converging regions into one component [4], [14]. For example, the different cortical regions, were compiled in one component covering a whole band of the cortex in the analysis with only 6 and 10 components (data not shown).

**Figure 1:**
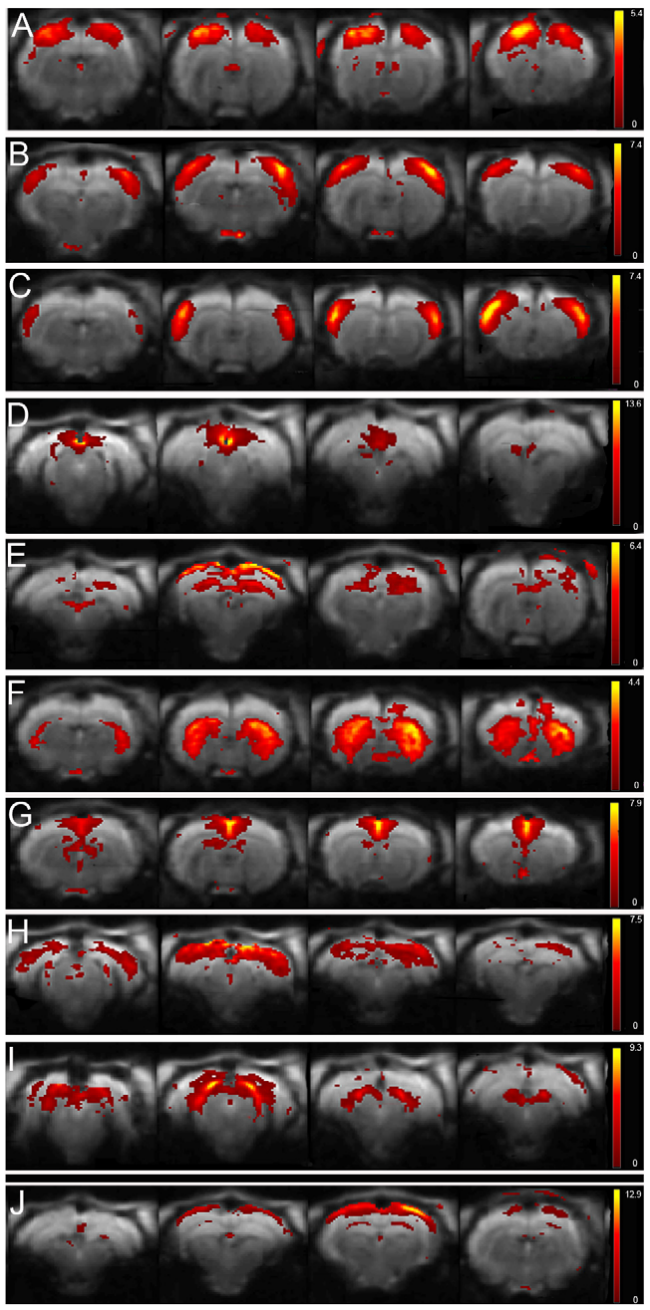
Functional connectivity maps resulting from 15 components ICA (GIFT) of rat rsfMRI. The figure shows 4 axial slices covering the anatomical area of each of the 9 mean components. The spatial colour-coded z-maps of these components are overlaid on the GE-EPI image. A higher z-score (yellow) represents a higher correlation between the time course of that voxel and the mean time course of this component. Mean components comprise (top-bottom) A) motorcortex, B) somatosensory cortex, C) auditory cortex, D) retrosplenial (dys)granular cortex, E) hippocampus, F) striatum, G) cingulate cortex, H) visual cortex and I) inferior colliculus; J) example of one slice effect.

Comparing the 15 components with known neuroanatomical regions, 9 meaningful circuits could be identified. These mean components are shown in Figure 1(A-I) and include motorcortex, somatosensory cortex, auditory cortex, retrosplenial (dys)granular cortex, hippocampus, striatum, cingulate cortex, visual cortex and colliculus inferior.

The remaining 6 components represented a small band located at the dorsal part of the cortex, near the brain surface, each confined to a single slice. One of these components is illustrated in Figure 1J. As anatomical regions can never be restricted to an arbitrary chosen single MRI slice, these components clearly point towards analysis induced artefacts. Moreover, these components remained identical throughout further analysis, independent of the chosen numbers of components or whether the subject was a rat or a mouse.

**Figure 2:**
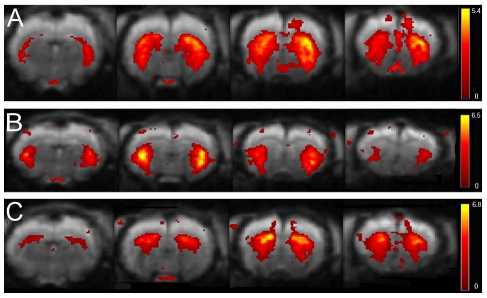
Striatal functional connectivity maps resulting from 15 and 40 components ICA (GIFT) of rat rsfMRI. The figure shows 4 axial slices of mean components, located at the striatum. The spatial colour-coded z-maps of these components are overlaid on the GE-EPI image. A higher z-score (yellow) represents a higher correlation between the time course of that voxel and the mean time course of this component. For the 15 component analysis (A) the striatum was shown confined in only one component but was divided over two components for the 40 component analysis (B & C).

The observed cortical functional connectivity maps of 40-component analysis were very similar, but some regions were split-up in different components. The striatum for example was divided in two components, one representing the ventrolateral part, the other representing the dorsomedial part of the striatum (shown in Figure 2), indicating that within the striatum these two regions could have slightly different LFF dynamics. Moreover, panel B in the figure shows that there is some similarity in the dynamics of the striatum and the somatosensory cortex. Also the colliculus inferior and cingulated cortex were separated in the 40 as compared to the 15 component analysis and the piriform cortex came out as an additional component.

Overall this resulted in an extension to 19 anatomically relevant components. The 6 artefactual components, as stated before, appeared very comparable in this analysis. Since the remaining components were either more diffuse, or only comprising a few voxels, it was impossible to co-localize these with the atlas.

### Mouse rsfMRI data

**Figure 3:**
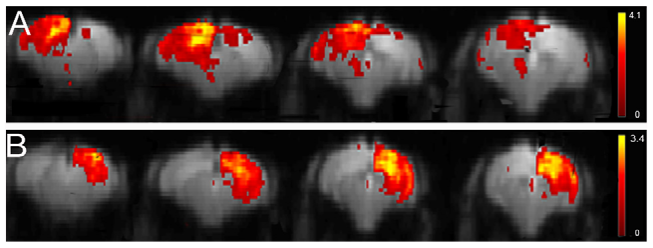
Cortical functional connectivity maps resulting from 15 components ICA (GIFT) of mouse rsfMRI. The figure shows 4 slices of 2 mean components, demonstrating the separation in the left (A) and right cortex (B). The spatial colour-coded z-maps (axial) of these components are overlaid on the GE-EPI image. A higher z-score (yellow) represents a higher correlation between the time course of that voxel and the mean time course of this component.

When comparing the 15 components mouse data to the rat rsfMRI data, some striking differences were observed. In mice, the entire cortex was unified in one single band covering somatosensory, auditory and visual cortices. A similar observation was made in the rat brain but only when using approximately 6 to 10 components instead of 15. A much more prominent difference was the observation that left and right cortex resorted in two separate components (shown in Figure 3), while the rat data always displayed clear bilateral networks for each part of the cortex.

Similar to the rat, the mouse rsfMRI resulted in two separate bilateral components representing the motorcortex and the striatum. Another component covered both cingulated cortex and (dys)granular cortex, which were present in two separate components in the rat data. The colliculus inferior component observed in rats could not be detected in mice.

Conversely, some relevant anatomical regions missing in the rat components were represented in one or more components in the mouse data: i.e.unilateral the entorhinal cortex and a bilateral piriform cortex component (only present in the 40 component analysis of rat data, also bilateral).

**Figure 4:**
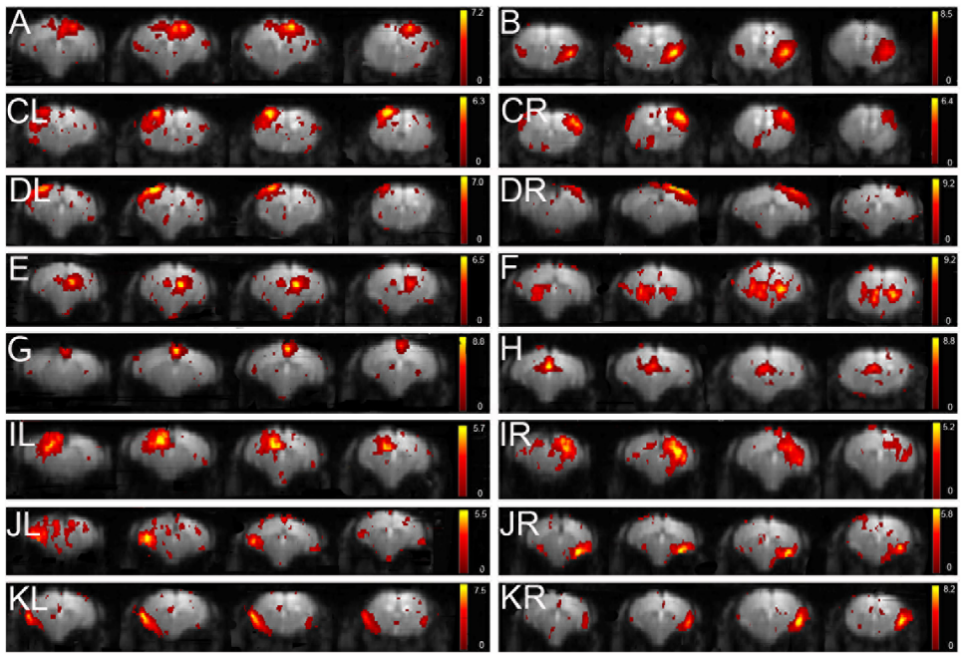
Functional connectivity maps resulting from 40 components ICA (GIFT) of mouse rsfMRI. The figure shows 4 axial slices of the 16 mean components. The spatial colour-coded z-maps of these components are overlaid on the GE-EPI image. A higher z-score (yellow) represents a higher correlation between the time course of that voxel and the mean time course for this component. Mean components comprise (top-bottom) A) motorcortex, B) piriform cortex, CL) left somatosensory cortex, CR) right somatosensory cortex, DL) left auditory cortex, DR) right auditory cortex, E) right hippocampus (dorsal), F) ventral hippocampus, G) retrosplenial (dys)granular cortex, H) cingulate cortex, IL) left visual cortex, IR) right visual cortex, JL) left medial entorhinal cortex, JR) right medial entorhinal cortex, KL) left lateral entorhinal cortex and KR) right lateral entorhinal cortex.

Figure 4 shows an overview of some (16) of the mouse components of the 40 component analysis including motorcortex, piriform cortex, left and right somatosensory cortex, left and right auditory cortex, right dorsal hippocampus, ventral hippocampus, retrosplenial (dys)granular cortex, cingulated cortex, left and right visual cortex, medial and lateral entorhinal cortex, both left and right.

**Figure 5:**
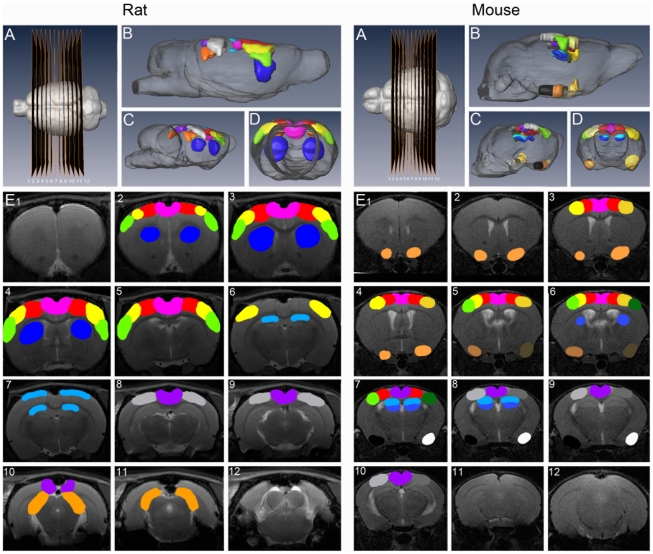
Schematic overview of the localization of different components shown in Figure 1 and 4. Left overview of the components resulting of the 15 component rat data analysis, right overview of the components resulting of the 40 component mouse data analysis. Both Panels A show the localization of the slices (1-12) from left to right. Panels B, C and D give a 3D surface rendered overview of the localization of the different components shown in figure 1 & 4 (B =  sagital, C = oblique, D =  axial view). Panels E gives this overview overlaid on RARE images. Colourcode: *Rat:* motorcortex (red), colliculus inferior (light orange), somatosensory cortex (yellow), auditory cortex (green), hippocampus (light blue), striatum (dark blue), retrosplenial (dys)granular cortex (purple), cingulate cortex (pink), visual cortex (grey). *Mice:* motorcortex (red), piriform cortex (orange), left somatosensory cortex (light yellow), right somatosensory cortex (dark yellow), left auditory cortex (light green), right auditory cortex (dark green), right dorsal hippocampus (light blue), ventral hippocampus (middle blue), retrosplenial (dys)granular cortex (purple), cingulate cortex (pink), left visual cortex (light grey) right visual cortex (dark grey), left medial entorhinal cortex (light brown), right medial entorhinal cortex (dark brown), left lateral entorhinal cortex and (white) right lateral entorhinal cortex (black).

Figure 5 shows the 3d surface rendering of the main components from the mice (40 comp. ICA) and rat (15 comp. ICA) rsfMRI to clarify their approximate mutual localization.

As shown in the figure, in this analysis the different parts of the mouse cortex are divided over different components. In contrary, a similar cortex components division could already be obtained in rats using a lower number of components (15). The same applies for cingulate and (dys) granular cortex, which in this analysis are presented as two different components compared to a single component in the 15 component analysis. In this analysis three more components as described before as artefactual were seen (9 in total). As for the rat data, the remainder components were either more diffuse, or only comprising a few voxels.

Table 1 gives an overview of the appearance of the main anatomical regions within each ICA analysis, comparing mouse and rat data for the 15 and 40 component analysis. The table was limited to the components cited as being anatomically meaningfull, based on literature [4], [14].

**Table 1:** Overview of the appearance of the main anatomical regions within each ICA analysis, comparing mouse and rat data for the 15 and 40 component analysis.

	Rat		Mouse	
	15	40	15	40
motorcortex	1 component	1 component	1 component	1 component
Somatosensory cortex (SSC)	1 component	1 component	2 unilateral components (left + right) covering SSC, AC and VC	2 components (left + right)
auditory cortex (AC)	1 component	1 component	2 unilateral components (left + right) covering SSC, AC and VC	2 components (left + right)
Retrosplenial (dys)granular cortex (RC)	1 component	1 component	1 component covering RC and CC	1 component
hippocampus	1 component	1 component	1 component	2 components (dorsal + ventral)
striatum	1 component	2 components	/	/
cingulate cortex (CC)	1 component	3 components	1 component covering RC and CC	1 component
visual cortex	1 component	2 components	2 unilateral components covering SSC, AC and VC	2 components (left + right)
inferior colliculus	1 component	6 components	/	/
piriform cortex	/	1 component	1 component	1component
entorhinal cortex	/	/	entorhinal cortex 1 component (right)	4 component left medial and lateral + Right medial and lateral)

### Reproducibility

#### Rat rsfMRI data

**Figure 6:**
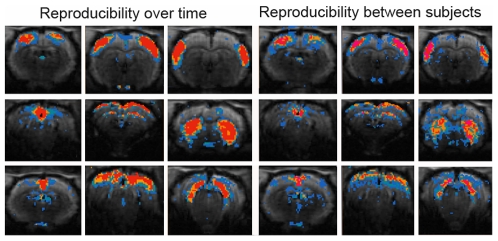
Reproducibility between sessions and between subjects of 15 components ICA (GIFT) of rat rsfMRI. Left: Pictures showing session cumulative score maps for 9 selected mean ICA rat components (central slice) over the different time points overlaid on the GE-EPI image. Colour-code: voxels with z-value higher than 1 for one time point (blue), two time points (blue-green), three time points (orange), four time points (red). Right: Pictures showing animal cumulative score maps for 9 selected ICA rat components (central slice) of the five rats overlaid on the GE-EPI image. Colour-code: voxels with z-value higher than 1 for one animal (blue), two animals (blue-green), three animals (orange), four animals (red), five animals (pink).

Because each of the five rats were scanned four times it was possible to visualize the reproducibility over time and between subjects (Figure 6 left and right, respectively). This analysis was done for the 9 anatomic relevant components of the 15 component analysis. The reproducibility over time map shows areas with voxels having a Z-value higher than 1 at 1, 2, 3 or all 4 repetitive time points in different colours. From this distribution it can be seen that the central part of each component shows overlap for all or at least three time points while voxels towards the border tend to represent data from a single time point. The figure shows that rsfMRI acquired during the same session or even with a week in between result in reproducible components.

Comparing components between subjects, demonstrates different regions for which the voxels have a Z-value higher than 1 for 1, 2, 3, 4 or 5 animals in different colours. Comparable with the intersession reproducibility, in this test the central part of each component overlaps for more animals. Voxels towards the border tend to represent data from a single animal. Visual comparison of both figures indicates that, as expected the *reproducibility over time* is better than the *reproducibility between animals.*

The same conclusion could be taken when the reproducibility assessment was repeated for the 40-components analysis (data not shown). Namely, the central part of each component was most reproducible both over time and between subjects.

#### Mouse rsfMRI data

For mice only two scans were acquired. If we repeated the same procedure for both 15 and 40-component data we could also conclude that the largest, central part of each component was reproducible for both scans.

**Figure 7:**
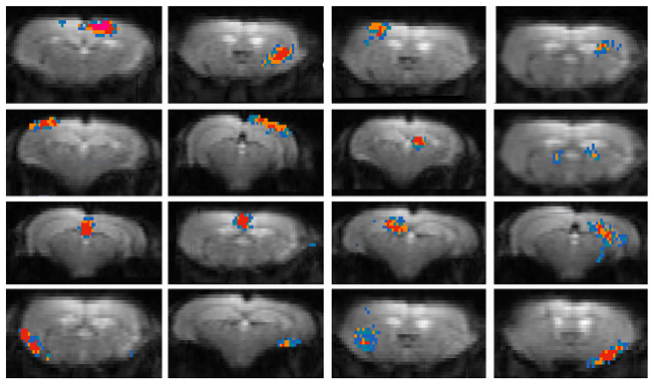
Reproducibility between subjects of 40 components ICA (GIFT) of mouse rsfMRI. Pictures showing animal cumulative score maps for 16 selected ICA mice components (central slice) of the nine mice overlaid on the GE-EPI image. Colour-code: voxels with z-value higher than 1 for five animals (blue), six animals (blue-green), seven animals (orange), eight animals (red), nine animals (pink).

To visualize the animal reproducibility the procedure was slightly adapted. For each subject the voxels were selected that were part of the component for both scans. Secondly, the same colours were used to visualize the results, here representing those voxels which were present in the same component for 5, 6, 7, 8 or all 9 mice. These figures are shown in Figure 7. It is important to mention that this method is more robust in comparing: the regions shown in the figure are only these which are at least reproducible for 5 animals, in contrast the comparable figure for rat data (Figure 6) also shows voxels which are present in only one animal. This results overall in smaller regions represented. It should also be mentioned that the motorcortex and piriform cortex, presented as bilateral components are only highly reproducible at one side, being the right side. Apparently the voxels represented at the left side show a larger spatial variation for the different animals. Also for the somatosensory cortex and visual cortex components it should be mentioned that the highly reproducible part of the components are more centrally located. If the figures are extended with 9 different colours showing all different numbers of animals the whole cortical part of the components is shown (data not given).

## Discussion

This study aimed at (1) implementing resting state functional connectivity measurements, currently increasingly used in human neuropathological research, in mice, a species that holds the widest variety of neurological disease models and (2) to compare the findings with those obtained in rats.

For the moment, most of the studies investigating functional connectivity in rats use data-analysis methods comprised to certain ROI's. In this study, however, it was chosen to work with a data-driven technique, called Independent Component Analysis. This technique is also frequently applied in clinical research to estimate functional connectivity of the brain. The technique allows data analysis without prior knowledge and gives the opportunity to investigate functional connectivity of the entire brain. It is important to mention that selection of anatomically relevant regions from the functional connectivity maps is still done by the investigator. Consequently, the interpretation of the networks remains subjective. One of the drawbacks of the technique is that the interpretation of the results is less straight forward than for ROI analysis. Moreover as our results indicate, the number of components chosen has an important influence on the observed functional connectivity maps.

First the rat data were analyzed using an ICA dividing the rat rsfMRI signal into 40 components, comparable with an earlier study applying ICA for rat data [14]. To test the effect of the number of components, a lower number (15) was arbitrarily chosen. This second analysis resulted in very similar components. Some regions, such as colliculus inferior and cingulate cortex, could be identified more accurately with this second analysis. For interspecies comparison the mouse data were analysed using the same numbers of components. Because we observed a bigger difference between both analyses for mice, we additionally performed an intermediate 30 component analysis. These data are not shown since the results appeared to be very similar to the 40 component analysis. Different cortical regions were compiled in one component for the 15 component analysis of mouse data, but not for the rat data. By reducing the number of components to 6 or 10, we could also observe for rat data the compilation of the cortical regions in one component.

Overall it is very important to mention that results of this type of analysis are dependent on both scanning and analysis parameters. For that reason results can only be compared in exactly comparable set-ups. We have tried to image and analyse the data of both species as comparable as possible. Although both rats and mice were scanned with the same MRI system, different RF acquisition coils were used to acquire optimal images in both species.

Moreover, size differences created the need for different slice thickness, resulting in a different voxel size. Yet we have tried to cover in both species a comparable area of the brain. We should take into account that not only the voxels but also the brain and the volume of the networks of the rats are larger.

The signal-to-noise ratio was slightly higher for the mice scans (87,92 vs. 67,09). Temporal signal-to-noise ratio's were equal for both species (p-value T-test  =  0,68). Partial volume effects are more important in the in-plane direction since several resting state networks are located side by side in this plane. Large voxels can result in a less prominent transition between networks if voxels contain signals compiled by the time course of different networks. Assuming that partial volume effects would be larger in rats, this is contradicted by differentiation of several cortical networks in rats located very close to each other.

Then in processing also a lot of parameters have to be taken into account, that could influence the outcome of the analysis. First of all, the smoothing kernel - which had a size of approximately 2 voxels [13], [32] - was slightly adapted for the mouse data but due to a rectangular matrix and square FOV this was not completely comparable for both species. Repeating the analysis with smoothing kernels exactly two-by-two voxels for both species resulted in components comparable to those presented in the paper. Also results with and without temporal filtering were compared. This was done using a band pass filter (0.01–0.1 Hz). In human research the limit of 0.1 Hz is used to exclude high frequency physiological fluctuations. It should be mentioned that due to the high cardiac and breathing rates in rodents the 0.1 Hz filter is insufficient [21] although it does not compromise the presented analysis strategy, because ICA can identify/isolate undersampled (aliased) processes.

We have chosen to apply this filter, in convergence with the ICA study of Hutchison et al. to compare the results. The results without filter were very comparable although more artefactual components were present in the non-filtered analyses, and some regions e.g. the hippocampus component could not be discerned. We also repeated the analyses with and without applying a spatial brain mask to the data. The effect of this mask was minimal, resulting in almost identical components. Furthermore ICA can be performed using different algorithms [33] and different numbers of components which has an important influence on the results, as proven in this paper.

An important difference between human MRI and small animal imaging is the need for anaesthesia to avoid movement. This is an important issue since both the BOLD response as well as the temporal correlation of LFFs between regions can be affected by anaesthesia [34]. We selected medetomidine of which it has been proven that it preserves functional connectivity [22] above all other options such as α-chloralose, isoflurane or ketamine/xylazine because of their toxicity, known deteriorating effect on functional connectivity or absence of information on the effect on resting state, respectively [35].

Interestingly, a clinical study reported recently that resting state data obtained under anaesthesia can have a significant diminishing effect on the fronto-parietal networks while functional connectivity at the early sensory cortices was relatively preserved. Related hereto in anaesthetised animals these sensory networks can be observed whereas the fronto-parietal networks (default mode network and executive control network), related to higher functions, remained undetected. This indicates that even if such networks are present in rodents they would hardly be assessable under anaesthesia [36].

In our study we discerned separate sensorimotor, visual and auditory networks comparable to what has been described in humans [4], [37]. The cingulate cortex, represented in both rodent species as a single component, is separated into a posterior and an anterior part in humans and belongs to the default mode network [4]. This human network also includes the medial prefrontal cortex (see Figure 1, component G).

The other regions within the human network (bilaterally the medial and lateral temporal lobe, the retrosplenial and lateral temporal cortex, precuneus and hippocampal formation [38]) could however not be resolved in rodents.

While in humans primary and secondary visual cortical areas are separated in two components [4], [37] both are restricted to one component in the rodent brain. This could be related to the important anatomical difference in the visual system resulting from the frontal versus lateral sight in humans versus rodents respectively. Moreover, in our rat study the visual cortex component was smaller as compared to the findings by Hutchison [14] and our mouse data. This difference could be explained by the reported visual cortex deficits [39] in albino rats like the Sprague-Dawley rats we used compared to the non albino Long Evans strain used by Hutchison and coworkers and the non albino (C57BL/6) mice we used.

Some regions, typically found in rodents, but not mentioned as being part of a resting state network in human research, e.g. the inferior colliculus, the entorhinal and the piriform cortex, were also found each in a separate left and right component. In rats, the entorhinal cortex comprises 4 regions (dorsolateral, dorsointermediate, ventral intermediate and medial) while in mice it only comprises a medial and lateral region which could be displayed in different components [40]. Because of this higher fragmentation of the rat brain structures it is possible that in rats all different parts have a slightly different temporal profile. This could explain why those parts of the cortex could be recognized for mice, but not for rats. The piriform cortex, while not discriminated in human studies, emerges in both rats and mice upon 40 component ICA analysis and probably reflects the more important role of olfaction in rodents as compared to humans.

Moreover, we described the existence of a component showing striatocortical connectivity which can be interesting for example in animal models for Parkinson's [41] and Huntington's disease [42] as in this pathology the projections between the aforementioned areas are affected and are a target for therapeutic strategies.

Most of the observed components were in agreement with another recent study using ICA rsfMRI on the rat brain [14] although some differences should be mentioned in the experimental setup: the use of anaesthesia (medetomidine vs. xylazine-ketamine and isoflurane), the slice direction (axial vs coronal) and the strain of rats (Sprague-Dawley vs. Long Evans). Different to our findings, most of their components showed a degree of laterality which might be due to the difference in applied anaesthesia as medetomidine better preserves functional connections [14].

The detection of unilateral components in mice compared to bilateral components for the same networks in rats indicate that the LFF of left and right cortices are different in mice while being similar in rats. This could possibly reflect a lower interhemispheric functional connectivity in mice. Since the same homotopic interhemispheric cortical connections are found in both rats and mice [37] there is probably no anatomical substance for this difference. It is known that functionally linked resting-state networks reflect the underlying structural connectivity, since most of the nodes of a single resting state network are interconnected with white matter tracts [43]. The same is true for these cortical regions, connected through the corpus callosum in both species.

Consequently the difference is located on a functional level rather than the underlying anatomical structure, indicating the need to study the temporal characteristics of the low frequency BOLD signal in the anaesthetized mouse brain and compare this with earlier findings of rats [10].

Moreover it would be interesting to study the differences between both species using a electrophysiological read-out. It is known that there is a strong correlation in LFFs measured with BOLD fMRI and fluctuations discerned in the power of the local field potential (LFP) signal as measured at different parts of the cortex in monkeys [16], [44]. Studying these LFPs in both rats and mice could give more insight in what was found here. Although we should mention that it is impossible to validate the results with the same study set-up since electrophysiological recordings can't be performed under medetomidine anesthesia.

Using different numbers of independent components revealed the need for higher number of components in order to discriminate different parts of the cortex in mice as compared to rats. This could reflect a more subtle difference in LFF between different neuronal networks in mice or might be linked to their smaller brain volume.

To gain more insight in what causes these differences there is a need to perform a parallel electrophysiological study in the future. Similarly, it could be anticipated that a differential use of the number of components might lead to detection of abnormalities in models linked to disease induced LFF differences between networks that might otherwise remain uncovered.

## Materials and Methods

### Ethics statement

All procedures were performed in accordance with the European guidelines for the care and use of laboratory animals (86/609/EEC) and were approved by the Committee on Animal Care and Use at the University of Antwerp, Belgium.

### Animals

Five male Sprague-Dawley rats (Charles River Laboratories, Wilmington, USA) (Body weight 454±60 g) of approximately 12 weeks and 9 male C57BL/6 mice (Charles River Laboratories, Wilmington, USA) (body weight 25±2 g) of approximately 13 weeks were imaged. Rats were imaged at 2 time points, with one week in between, and at each time point two consecutive resting state scans were acquired. Mice were imaged at one time point, at which two consecutive resting state scans were acquired.

Taking into account the age of sexual maturity and neuronal development of both species we can assume that the developmental age at the time of scanning is comparable for rats and mice [45]–[47].

### rsfMRI procedure

#### Rats

A rectal thermistore was inserted to monitor the body temperature and keep it at (37.0±0.5)°C by means of a feedback-controlled warm air circuitry (MR-compatible Small Animal Heating System, SA Instruments, Inc.).

Breathing rate, heart rate and blood oxygen saturation were monitored using a pulse oximeter positioned at the hind limb and a pressure sensitive sensor under the rat (MR-compatible Small Animal Monitoring and Gating System, SA Instruments, Inc).

During handling (weighing and immobilization) rats were anaesthetized with isoflurane (2% IsoFlo, Abott, Illinois, USA) administered in a mixture of 30% O_2_ and 70% N_2_. During the resting state experiment, animals were sedated using medetomidine (Domitor, Pfizer, Karlsruhe, Germany). First a bolus of 0.05 mg/kg was injected subcutaneously and isoflurane was discontinued after 5 minutes.

A continuous infusion of medetomidine (0.1 mg/kg/h) was started 15 minutes after bolus injection [48]. After the scanning procedure, medetomidine was antagonized by an injection of atipamezole (0.1 mg/kg) (Antisedan, Pfizer, Karlsruhe, Germany).

Imaging was done on a 9.4T Biospec scanner (Bruker, Ettlingen, Germany). Images were acquired with a Bruker linear transmit volume coil and a parallel receive surface array designed for rat head MRI. First three orthogonal Turbo RARE T2-weighted images were acquired to uniform the slice positioning (TR 2500 ms, effective TE 33 ms, 15 slices, 1 mm). Then the resting state data-sets were acquired using single shot gradient echo EPI (Echo Planar Imaging) with TR 2000 ms and TE 16 ms. Twelve axial slices of 1 mm and a gap of 0.1 mm were recorded with a FOV of (30×30) mm^2^ and matrix size of 128×128 resulting in voxel dimensions of (0.23×0.23×1) mm^3^. The used bandwidth was 400 kHz (3125 Hz/voxel). Each of the rsfMRI data comprised 150 repetitions, resulting in a scanning time of 5 minutes for each rsfMRI dataset.

#### Mice

The same monitoring procedures were followed during the mice scans and the same principle was used to sedate the mice. A bolus of 0.3 mg/kg medetomidine was followed by continuous infusion of 0.6 mg/kg/h [30].

Imaging was also done on a 9.4T Biospec scanner (Bruker, Ettlingen, Germany). Images were acquired with a standard Bruker crosscoil set-up using a quadrature volume coil and a quadrature surface coil for mice. First three orthogonal Turbo RARE T2-weighted images were acquired to uniform the slice positioning (TR 2500 ms, effective TE 33 ms, 9 slices, 0.5 mm).

Then the resting state data-sets were acquired using single shot gradient echo EPI with TR 2000 ms and TE 15 ms. Twelve axial slices of 0.4 mm and a gap of 0.1 mm were recorded with a FOV of (20×20) mm^2^ and matrix size of 128×64 resulting in voxel dimensions of (0.16×0.31×0.4) mm^3^. The used bandwidth was 400 kHz (3125 Hz/voxel in read/6250 Hz/voxel in phase encoding). Hundred fifty repetitions were taken, resulting in a 5 minute scanning time for each rsfMRI dataset.

### Preprocessing

Preprocessing was done in SPM8 (http://www.fil.ion.ucl.ac.uk/spm/software/spm8/). A common protocol for preprocessing of fMRI data was followed. First, all images within each session were realigned to the first image. This was done using a least squares approach and a 6 parameter (rigid body) spatial transformation. Second, all datasets were normalized. The first step of the normalisation is a global 12-parameter affine transformation based on the maximalization of the product of the likelihood function (derived from the residual squared difference) and the prior function (which is based on the probability of obtaining a particular set of zooms and shears) [49]. The affine registration is followed by estimating nonlinear deformations, whereby the deformations are defined by a linear combination of three dimensional discrete cosine transform (DCT) basis functions [50]. The matching involved simultaneously minimising the membrane energies of the deformation fields and the residual squared difference between the images and template (which is here the rsfMRI images of the first animal). Finally, in plane smoothing was done using a Gaussian kernel with Full width at half maximum of (0.4×0.4) mm^2^ for the rat images and (0.3×0.4) mm^2^ for the mice images. A band pass filter (0.01 Hz–0.1 Hz) was applied to the temporal data to rule out low frequency noise.

### Processing

To estimate functional connectivity, ICA was performed, using GIFT (Group ICA of fMRI toolbox: http://icatb.sourceforge.net/), working in Matlab2008 (www.mathworks.com), and implementing spatial ICA, indicating that the sources are estimated as being statistically spatially independent. The GIFT toolbox is especially designed to analyze group rsfMRI data, and works in three steps.

First, a data reduction is done, by principal component analysis. This was done in two steps where first the functional data were reduced, followed by concatenation of the data in groups. Second, a group ICA is performed using the Infomax algorithm. Last, a back reconstruction of the data to single subject independent components and time courses was done.

To investigate the effect of the number of components, ICA of rat rsfMRI data was repeated several times with different preset component numbers.

The analysis was repeated, setting the number at 6, 10, 15 and 40 components. The ICA on the rsfMRI data of mice was assessed with a preset of 15, 30 and 40 components.

## References

[1] Biswal B, Yetkin FZ, Haughton VM, Hyde JS (1995). Functional connectivity in the motor cortex of resting human brain using echo-planar MRI.. Magn Reson Med.

[2] van den Heuvel MP, Hulshoff Pol HE (2010). Exploring the brain network: a review on resting-state fMRI functional connectivity.. Eur Neuropsychopharmacol.

[3] Damoiseaux JS, Beckmann CF, Arigita EJ, Barkhof F, Scheltens P (2008). Reduced resting-state brain activity in the “default network” in normal aging.. Cereb Cortex.

[4] Damoiseaux JS, Rombouts SA, Barkhof F, Scheltens P, Stam CJ (2006). Consistent resting-state networks across healthy subjects.. Proc Natl Acad Sci U S A.

[5] Greicius MD, Flores BH, Menon V, Glover GH, Solvason HB (2007). Resting-state functional connectivity in major depression: abnormally increased contributions from subgenual cingulate cortex and thalamus.. Biol Psychiatry.

[6] Rombouts SA, Damoiseaux JS, Goekoop R, Barkhof F, Scheltens P (2009). Model-free group analysis shows altered BOLD FMRI networks in dementia.. Hum Brain Mapp.

[7] Bluhm RL, Miller J, Lanius RA, Osuch EA, Boksman K (2007). Spontaneous low-frequency fluctuations in the BOLD signal in schizophrenic patients: anomalies in the default network.. Schizophr Bull.

[8] Wise RG, Ide K, Poulin MJ, Tracey I (2004). Resting fluctuations in arterial carbon dioxide induce significant low frequency variations in BOLD signal.. Neuroimage.

[9] Gusnard DA, Raichle ME, Raichle ME (2001). Searching for a baseline: functional imaging and the resting human brain.. Nat Rev Neurosci.

[10] Kannurpatti SS, Biswal BB, Kim YR, Rosen BR (2008). Spatio-temporal characteristics of low-frequency BOLD signal fluctuations in isoflurane-anesthetized rat brain.. Neuroimage.

[11] Zhao F, Zhao T, Zhou L, Wu Q, Hu X (2008). BOLD study of stimulation-induced neural activity and resting-state connectivity in medetomidine-sedated rat.. Neuroimage.

[12] Biswal BB, Kannurpatti SS (2009). Resting-state functional connectivity in animal models: modulations by exsanguination.. Methods Mol Biol.

[13] van Meer MP, van der Marel K, Otte WM, Berkelbach van der Sprenkel JW (2010). Correspondence between altered functional and structural connectivity in the contralesional sensorimotor cortex after unilateral stroke in rats: a combined resting-state functional MRI and manganese-enhanced MRI study.. J Cereb Blood Flow Metab.

[14] Hutchison RM, Mirsattari SM, Jones CK, Gati JS, Leung LS (2010). Functional networks in the anesthetized rat brain revealed by independent component analysis of resting-state FMRI.. J Neurophysiol.

[15] Vincent JL, Patel GH, Fox MD, Snyder AZ, Baker JT (2007). Intrinsic functional architecture in the anaesthetized monkey brain.. Nature.

[16] Shmuel A, Leopold DA (2008). Neuronal correlates of spontaneous fluctuations in fMRI signals in monkey visual cortex: Implications for functional connectivity at rest.. Hum Brain Mapp.

[17] Moeller S, Nallasamy N, Tsao DY, Freiwald WA (2009). Functional connectivity of the macaque brain across stimulus and arousal states.. J Neurosci.

[18] Teichert T, Grinband J, Hirsch J, Ferrera VP (2010). Effects of heartbeat and respiration on macaque fMRI: implications for functional connectivity.. Neuropsychologia.

[19] Bifone A, Gozzi A, Schwarz AJ (2010). Functional connectivity in the rat brain: a complex network approach.. Magn Reson Imaging.

[20] Magnuson M, Majeed W, Keilholz SD (2010). Functional connectivity in blood oxygenation level-dependent and cerebral blood volume-weighted resting state functional magnetic resonance imaging in the rat brain.. J Magn Reson Imaging.

[21] Kalthoff D, Seehafer JU, Po C, Wiedermann D, Hoehn M (2010). Functional connectivity in the rat at 11.7T: Impact of physiological noise in resting state fMRI.. Neuroimage Epub ahead of print.

[22] Pawela CP, Biswal BB, Hudetz AG, Schulte ML, Li R (2009). A protocol for use of medetomidine anesthesia in rats for extended studies using task-induced BOLD contrast and resting-state functional connectivity.. Neuroimage.

[23] Pawela CP, Biswal BB, Cho YR, Kao DS, Li R (2008). Resting-state functional connectivity of the rat brain.. Magn Reson Med.

[24] Pawela CP, Biswal BB, Hudetz AG, Li R, Jones SR (2010). Interhemispheric neuroplasticity following limb deafferentation detected by resting-state functional connectivity magnetic resonance imaging (fcMRI) and functional magnetic resonance imaging (fMRI).. Neuroimage.

[25] Mohammadi B, Kollewe K, Samii A, Krampfl K, Dengler R (2009). Decreased brain activation to tongue movements in amyotrophic lateral sclerosis with bulbar involvement but not Kennedy syndrome.. J Neurol.

[26] Zhou Y, Shu N, Liu Y, Song M, Hao Y (2008). Altered resting-state functional connectivity and anatomical connectivity of hippocampus in schizophrenia.. Schizophr Res.

[27] van de Ven V, Formisano E, Prvulovic D, Roeder CH, Linden DE (2004). Functional connectivity as revealed by spatial independent component analysis of fMRI measurements during rest.. Hum Brain Mapp.

[28] Ahrens ET, Dubowitz DJ (2001). Peripheral somatosensory fMRI in mouse at 11.7 T. NMR Biomed.

[29] Nair G, Duong TQ (2004). Echo-planar BOLD fMRI of mice on a narrow-bore 9.4 T magnet.. Magn Reson Med.

[30] Adamczak JM, Farr TD, Seehafer JU, Kalthoff D, Hoehn M (2010). High field BOLD response to forepaw stimulation in the mouse.. Neuroimage.

[31] Van Der Linden A, van Camp N, Ramos-Cabrer P, Hoehn M (2007). Current status of functional MRI on small animals: application to physiology, pathophysiology, and cognition.. NMR Biomed.

[32] Majeed W, Magnuson M, Keilholz SD (2009). Spatiotemporal dynamics of low frequency fluctuations in BOLD fMRI of the rat.. J Magn Reson Imaging.

[33] Hyvarinen A, Oja E (2000). Independent component analysis: algorithms and applications.. Neural Netw.

[34] Williams KA, Magnuson M, Majeed W, Laconte SM, Peltier SJ (2010). Comparison of alpha-chloralose, medetomidine and isoflurane anesthesia for functional connectivity mapping in the rat.. Magn Reson Imaging.

[35] Silverman J, Muir WW (1993). A review of laboratory animal anesthesia with chloral hydrate and chloralose.. Lab Anim Sci.

[36] Boveroux P, Vanhaudenhuyse A, Bruno MA, Noirhomme Q, Lauwick S (2010). Breakdown of within- and between-network Resting State Functional Magnetic Resonance Imaging Connectivity during Propofol-induced Loss of Consciousness.. Anesthesiology.

[37] Beckmann CF, DeLuca M, Devlin JT, Smith SM (2005). Investigations into resting-state connectivity using independent component analysis.. Philos Trans R Soc Lond B Biol Sci.

[38] Raichle ME, Snyder AZ (2007). A default mode of brain function: a brief history of an evolving idea.. Neuroimage.

[39] Diykov DG (2005). Visual Cortex Defects in Albinos.. The Internet Journal of Ophthalmology and Visual Science.

[40] Paxinos G, Watson C (1982). T*he Rat Brain in Stereotaxic Coordinates*.

[41] Braak H, Sastre M, Del TK (2007). Development of alpha-synuclein immunoreactive astrocytes in the forebrain parallels stages of intraneuronal pathology in sporadic Parkinson's disease.. Acta Neuropathol.

[42] Walker FO (2007). Huntington's Disease.. Semin Neurol.

[43] van den Heuvel MP, Mandl RC, Kahn RS, Hulshoff Pol HE (2009). Functionally linked resting-state networks reflect the underlying structural connectivity architecture of the human brain.. Hum Brain Mapp.

[44] Scholvinck ML, Maier A, Ye FQ, Duyn JH, Leopold DA (2010). Neural basis of global resting-state fMRI activity.. Proc Natl Acad Sci U S A.

[45] Adams N, Boice R (1983). A longitudinal study of dominance in an outdoor colony of domestic rats.. J Comp Psych.

[46] Fox JG, Barthold SW, Davisson MT, Newcomer CE, Quimby FW (2007). The mouse in Biomedical Research..

[47] Clancy B, Finlay BL, Darlington RB, Anand KJ (2007). Extrapolating brain development from experimental species to humans.. Neurotoxicology.

[48] Weber R, Ramos-Cabrer P, Wiedermann D, van Camp N, Hoehn M (2006). A fully noninvasive and robust experimental protocol for longitudinal fMRI studies in the rat.. Neuroimage.

[49] Ashburner J, Neelin P, Collins DL, Evans A, Friston K (1997). Incorporating prior knowledge into image registration.. Neuroimage.

[50] Buchel C, Wise RJ, Mummery CJ, Poline JB, Friston KJ (1996). Nonlinear regression in parametric activation studies.. Neuroimage.

